# Genetic Reconstruction of Protozoan rRNA Decoding Sites Provides a Rationale for Paromomycin Activity against *Leishmania* and *Trypanosoma*

**DOI:** 10.1371/journal.pntd.0001161

**Published:** 2011-05-24

**Authors:** Sven N. Hobbie, Marcel Kaiser, Sebastian Schmidt, Dmitri Shcherbakov, Tanja Janusic, Reto Brun, Erik C. Böttger

**Affiliations:** 1 Institute of Medical Microbiology, University of Zurich, Zurich, Switzerland; 2 Medical Parasitology and Infection Biology, Swiss Tropical and Public Health Institute, Basel, Switzerland; 3 University of Basel, Basel, Switzerland; McGill University, Canada

## Abstract

Aminoglycoside antibiotics target the ribosomal decoding A-site and are active against a broad spectrum of bacteria. These compounds bind to a highly conserved stem-loop-stem structure in helix 44 of bacterial 16S rRNA. One particular aminoglycoside, paromomycin, also shows potent antiprotozoal activity and is used for the treatment of parasitic infections, e.g. by *Leishmania* spp. The precise drug target is, however, unclear; in particular whether aminoglycoside antibiotics target the cytosolic and/or the mitochondrial protozoan ribosome. To establish an experimental model for the study of protozoan decoding-site function, we constructed bacterial chimeric ribosomes where the central part of bacterial 16S rRNA helix 44 has been replaced by the corresponding *Leishmania* and *Trypanosoma* rRNA sequences. Relating the results from in-vitro ribosomal assays to that of in-vivo aminoglycoside activity against *Trypanosoma brucei*, as assessed in cell cultures and in a mouse model of infection, we conclude that aminoglycosides affect cytosolic translation while the mitochondrial ribosome of trypanosomes is not a target for aminoglycoside antibiotics.

## Author Summary

Rational design of novel therapeutics relies on the knowledge and understanding of potential drug targets. Historically, the majority of therapeutics have not been rationally designed, but empirically discovered. Paromomycin, an aminoglycoside with antibacterial activity, has been found to show considerable activity against leishmaniasis, a disease caused by the protozoan parasite *Leishmania*. However, the mechanisms of aminoglycoside action against protozoan parasites have in part remained unclear. In this study we demonstrate that the cytosolic ribosome is the preferred drug target, and that the mitochondrial ribosome does not contribute to the antiprotozoal activity of aminoglycosides. As the cytosolic ribosome of *Trypanosoma*, the causative agent of sleeping sickness and Chagas disease, resembles that of *Leishmania*, we tested the efficacy of paromomycin against *Trypanosoma*. We found that paromomycin not only inhibits the growth of *Trypanosoma* in culture, but also suppresses trypanosomiasis in a mouse infection model. Our results point to the cytosolic ribosome as a promising drug target for antiprotozoal drug development.

## Introduction

Aminoglycoside antibiotics show broad-spectrum antibacterial activity and are a common choice for treatment of serious infections due to gram-negative bacilli, including endocarditis, sepsis, pneumonia, and pyelonephritis [1]. Among the aminoglycoside antibiotics, paromomycin has also been shown to be effective against some protozoa and cestodes. The cost of paromomycin is low, making it a particular good drug candidate in countries that carry a burden of high parasitic infection rates. While paromomycin is out of use as an antibacterial, it is marketed as an oral treatment for amoebiasis and giardiasis. Paromomycin is also used in combination therapy as a topical treatment for cutaneous leishmaniasis [2]. Recently, paromomycin was licensed as a treatment for visceral leishmaniasis, the most severe form of leishmaniasis (reviewed in [3]).

Aminoglycosides exert their antibacterial activity by binding to a highly conserved region in helix 44 of bacterial 16S-rRNA [4]. We have previously reconstructed the drug target site of protozoan cytosolic ribosomes in chimeric bacterial ribosomes to demonstrate that the decoding site of cytosolic *Leishmania* ribosomes is susceptible to paromomycin but not to various other aminoglycosides [5]. These results have been recently confirmed by studies that showed specific paromomycin binding to the decoding site of *Leishmania* cytosolic ribosomes by surface plasmon resonance analysis [6].

While these studies have collectively provided a molecular rationale for the antileishmanial activity of paromomycin, these findings did not address whether in addition the *Leishmania* mitochondrial ribosome is targeted by aminoglycosides. Recent data have demonstrated that mitochondrial translation is essential for both the procyclic and the bloodstream form of *Trypanosoma brucei* and that consequently mitochondrial protein synthesis may represent an important drug target throughout the life cycle of trypanosomes [7]. There is, however, some inconsistency in the literature with regards to the effect of paromomycin on mitochondrial protein synthesis in *Leishmania*. For instance Maarouf et al. [8] have reported that paromomycin interferes with mitochondrial protein synthesis in *Leishmania*, whereas Horvath et al. [9] found no effect of paromomycin on mitochondrial translation.

**Figure 1:**
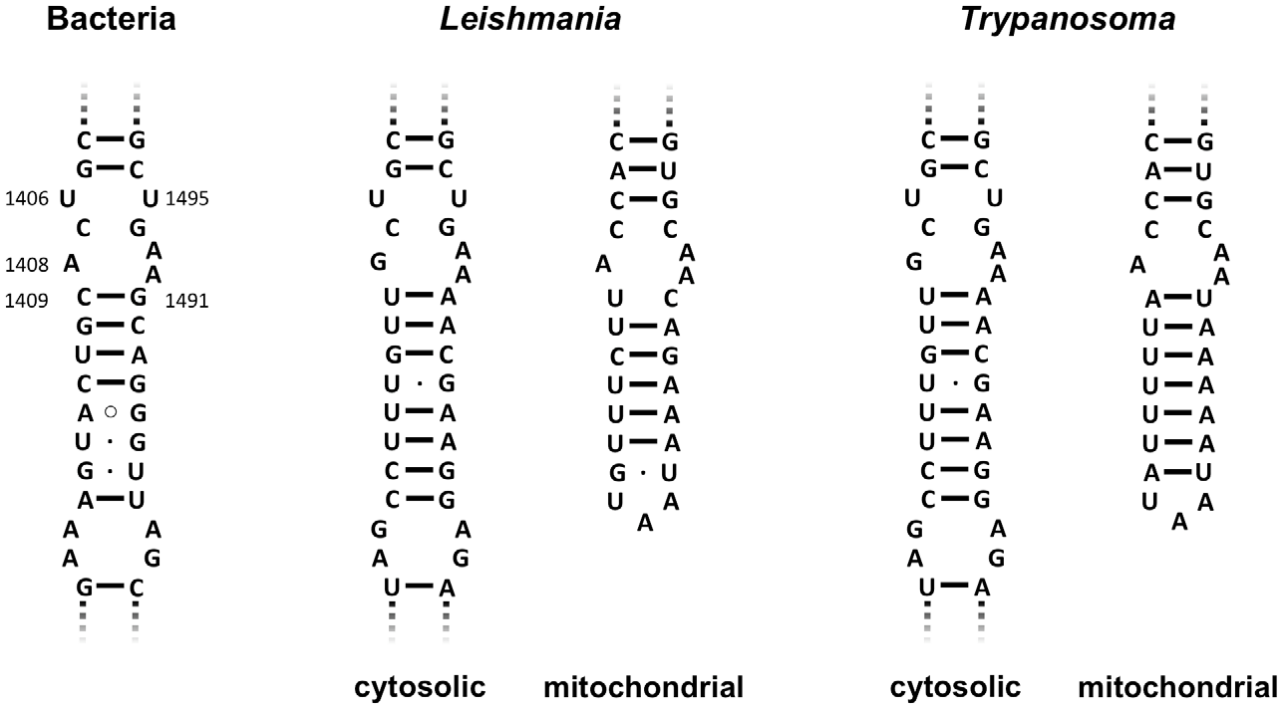
Secondary-structure comparison of small-subunit rRNA sequences. Phylogenetic comparison of the rRNA helices that correspond to the proximal portion of helix 44 in bacterial 16S rRNA, which is an integral part of the ribosomal decoding site.

Structural analysis of the *Leishmania* mitochondrial ribosome has revealed a remarkable morphologic similarity to the eubacterial ribosome [10]. However, the homolog of bacterial 16S rRNA helix 44 in trypanosome mitochondria is truncated in comparison to its bacterial counterpart, although the proximal part constituting the decoding site and the aminoglycoside-binding site is fully retained (Fig. 1). This is not surprising as the ribosomal decoding site is one of the most important catalytic domains within the ribosome, and it is universally conserved across all phylogenetic domains of life including organelles. At the same time, the mitochondrial rRNA of trypanosomes carries unique signatures within its decoding site sequence. Not only is this rRNA motif significantly different from bacterial 16S rRNA, it also shows a considerable sequence difference between the two closely related genera *Leishmania* and *Trypanosoma* (Fig. 1). The distinctive structural features of the mitochondrial decoding site make it difficult to predict its functional susceptibility to compounds that bind to the bacterial decoding site.

Here we reconstructed the mitochondrial decoding sites of *Leishmania* and *Trypanosoma* in bacterial ribosomes to analyze their susceptibility to aminoglycoside antibiotics and to allow for a comprehensive evaluation of the therapeutic potential of this class of drugs against trypanosome parasites. Based on the susceptibility pattern of chimeric ribosomes mimicking the ribosomal decoding sites of *Trypanosoma*, we assessed the antiprotozoal activity of paromomycin in cultures of *T. brucei* and in a mouse model of infection.

## Materials and Methods

### Ethics statement

Animal experiments were carried out in compliance with Swiss federal law (TSchG) and cantonal by-laws (TSchV Basel-Stadt). All protocols and procedures were reviewed and approved by the local veterinary authorities of the Kanton Basel-Stadt (Permit Number: 739).

### RNA secondary structure alignment

All rRNA nucleotides discussed in this study are numbered according to their homologous position in *E. coli* 16S rRNA. 9S rRNA genes of *L. donovani*, *L. major*, *L. amazonensis*, *L. tarentolae*, *T. brucei*, and *T. cruzi* (Genbank accession numbers FJ416603, EU140338, HM439238, M10126, M94286, and DQ343645, respectively) were used for rRNA sequence alignments of the mitochondrial decoding sites. 18S rRNA genes of *L. donovani*, *L. major*, *L. mexicana*, *L. amazonensis*, *L. braziliensis*, *L. tarentolae*, *T. brucei*, and *T. cruzi* (accession numbers FR799614, FR796423, GQ332360, GQ332354, GQ332355, M84225 AL929603, and FJ001665, respectively) were used for rRNA sequence alignments of the cytosolic decoding sites (Fig. S1).

### Construction of chimeric ribosomes

Recombinant *Mycobacterium smegmatis* strains with chimeric ribosomes were constructed by previously described gene replacement procedures. 1) rRNA fragments coding for mutant rRNA were generated by PCR mutagenesis and cloned into a suitable vector to result in chromosomal gene replacement via homologous recombination with donor DNA by selection of the mutant genotype [11]. 2) Plasmid replacement in a Δ*rrn* knockout strain of *M. smegmatis* mc^2^-155. DNA sequences coding for chimeric rRNA were generated by PCR, cloned into a plasmid coding for a full rRNA operon, and used to replace the wild-type rRNA sequence in *M. smegmatis* by means of plasmid exchange [5]. Strains and plasmids used in this study are listed in Tables S1 and S2. Successful rRNA replacement was controlled by sequence analysis.

### Drug susceptibility of chimeric ribosomes

Recombinant *M. smegmatis* strains were studied for susceptibility to paromomycin, neomycin, gentamicin, and tobramycin (Sigma Aldrich). Minimal inhibitory concentrations (MIC) were determined by broth microdilution tests as described previously [12]. The gentamicin used in this study is a mixture of gentamicin C_1_, gentamicin C_1a_, and gentamicin C_2_ in a 45∶35∶30 ratio. The chemical structures of aminoglycoside antibiotics are provided as Fig. S2.

### In vitro assays

Bloodstream forms of *Trypanosoma brucei rhodesiense* STIB900 and axenic amastigote forms of *L. donovani* (MHOM-ET-67/L82) were used for the in vitro assays. Cytotoxicity was assessed with rat skeletal myoblasts (L6 cells). IC_50_ values were obtained using a resazurin-based assay (alamarBlue) as described earlier [13]. The following compounds were used as standards: melarsoprol (*T.b.rhodesiense*), miltefosine (*L. donovani*), and podophyllotoxin (L6 cells).

Test compounds were dissolved in distilled water. Following initial experiments the assay duration time was extended to 96 hrs, 120 hrs and 144 hours for *L. donovani* to demonstrate in vitro activity of paromomycin. For the assays with extended duration a subculture was prepared after 72 hrs by transferring 10 µl of the initial assay culture to 100 µl fresh medium containing the corresponding compound concentration.

### In vivo efficacy against *T.b.brucei* STIB795

Groups of 4 female NMRI mice (Harlan Netherlands) of 22–25 g were infected by the intraperitoneal route with 10^4^ bloodstream form of *T. brucei brucei* STIB795 per mouse. Paromomycin was formulated in physiological saline solution and administered i.p. in a volume of 10 ml kg^−1^.Treatment was initiated on day 3 post-infection. The mice were monitored for parasitaemia 24 hrs after the last treatment (day 7) and again on day 10 and 14 post-infection. Once parasitaemia exceeded 10^7^/ml the animals were euthanized. The day of relapse was used as endpoint.

## Results

**Figure 2:**
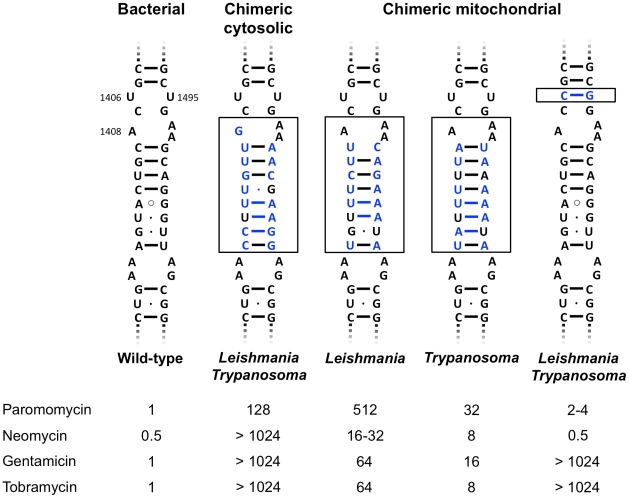
Aminoglycoside susceptibility of rRNA chimeras. Aminoglycoside susceptibility is expressed as minimal inhibitory concentrations (MIC, µg/mL). The protozoan sequence part is boxed with the protozoan-specific nucleotides colored in blue.

The parasitic trypanosomes (Kinetoplastida: Trypanosomatidae) include the genera *Trypanosoma* and *Leishmania*. We have previously reconstructed the aminoglycoside drug binding pocket, i.e. the decoding A-site, of trypanosome cytosolic ribosomes in bacteria [5]. To do so, we have replaced the proximal stem of helix 44 in bacterial 16S rRNA, i.e. nucleotides homologous to positions 1408–1416 and 1484–1491 in bacterial 16S rRNA, with the corresponding cytosolic rRNA sequences present in *Leishmania* and *Trypanosoma* species (Fig. 1 and 2). In the same study, we demonstrated the specific activity of paromomycin against *Leishmania* and *Trypanosoma* chimeric cytosolic ribosomes [5].

For a comprehensive analysis of trypanosomal susceptibility to 2-deoxystreptamines, we reconstructed the mitochondrial decoding A-sites of *Leishmania* and *Trypanosoma* in bacterial ribosomes. The mitochondrial rRNA sequence, and in particular the decoding A-site is considerably different between *Leishmania* and *Trypanosoma* (Fig. S1). Base pair 1409—1491, which is considered key in aminoglycoside susceptibility, is characterized by an U•C interaction in *Leishmania* versus an A—U base pairing in *Trypanosoma* (Fig. 1). Furthermore, the mitochondrial A-site of *Leishmania* and *Trypanosoma* exhibits a unique 1406C—1495G base pairing. This is in contrast to the cytosolic decoding A-site, which shows the typical 1406U—1495U interaction that is universally conserved across the three domains of life including organelles. Attempts to reconstruct the entire mitochondrial trypanosome A-site in bacteria as a 27-nucleotide helix comprising residues 1404–1416 and 1484–1497 were unsuccessful. We thus reconstructed the trypanosome homologue of helix 44 as two mutant chimeric ribosomes: one with a 19-nucleotide helix of *Leishmania* and *Trypanosoma* mitochondrial rRNA corresponding to *M. smegmatis* positions 1408–1416 and 1484–1493, and one mutant recombinant carrying the characteristic trypanosome mitoribosomal 1406C—1495G pair (Fig. 2).

Studying the bacterial recombinants revealed that the mitochondrial chimeras with the 19-nucleotide replacement are susceptible to compounds with a 6′-amino substituent, such as neomycin, gentamicin, and tobramycin, and less susceptible to paromomycin, which carries a 6′-hydroxy substituent (Fig. 2; the chemical structures of the tested 2-deoxystreptamines are provided in Fig. S2). In addition, the *Trypanosoma* 19-nucleotide chimera was found to be generally more susceptible to aminoglycosides than its *Leishmania* counterpart reflecting the canonical base pair interaction between residues 1409 and 1491 (Fig. 2). Recombinants carrying the 1406C—1495G pair were susceptible to the 4,5-disubstituted aminoglycosides neomycin and paromomycin, but highly resistant to the 4,6-disubstituted aminoglycosides gentamicin and tobramycin (Fig. 2). From the combined results we conclude that among the aminoglycosides tested, neomycin has the highest activity against the protozoan mitoribosomes, while gentamicin and tobramycin are virtually inactive.

The finding that the *Trypanosoma* ribosomal decoding sites are at least as susceptible to paromomycin as those of *Leishmania* ribosomes prompted us to determine the efficacy of aminoglycosides against *Trypanosoma* in a cell culture assay. We found that paromomycin suppressed growth of *Trypanosoma brucei rhodesiense* in vitro (Table 1). Compared to *T. brucei rhodesiense*, IC_50_ determinations of paromomycin in *L. donovani* required extended assay durations. Notably, the 50% inhibitory concentration (IC_50_) of paromomycin was more than tenfold lower with *T. brucei* than with *L. donovani* (Table 1). To assess whether mitochondrial protein synthesis is involved in paromomycin's potent antiprotozoal activity, we also studied the efficacy of neomycin against *T. brucei*. Neomycin shows significant activity against *Trypanosoma* mitochondrial recombinant ribosomes but little activity against *Trypanosoma* cytosolic chimeras (Fig. 2). Thus, if trypanosome mitochondria were to be targeted by aminoglycosides, we would expect neomycin to show activity against *T. brucei*. However, compared to paromomycin, neomycin had no effect on the growth of *T. brucei* (Table 1). Tobramycin, an aminoglycoside that is inactive against both cytosolic and mitochondrial chimeric ribosomes (Fig. 2), was used as a control and had no effect on trypanosome growth in culture (Table 1).

**Table 1:** Antiprotozoal activity of selected aminoglycoside antibiotics in cell culture.

		IC_50_ (µg/ml)	
	Assay duration (h)	*T. brucei rhodesiense*	*L. donovani*
Paromomycin	72	6.2	>90
	96	ND^a^	75.8
	120	ND	62.2
	144	3.3	54.5
Neomycin	72	>60	ND
Tobramycin	72	>60	ND

a ND, not determined.

Based on the above findings we wished to study the efficacy of paromomycin in the *T. brucei brucei* STIB795 mouse model of infection. Paromomycin suppressed the growth of *T. brucei* in vivo and increased the mean survival time even at the lowest dose regimen of 100 mg per kg of body weight given for 4 days (Table 2). However, high concentrations of paromomycin did not fully eradicate the parasite in the mouse infection model, as parasitemia relapsed once treatment was stopped.

**Table 2:** In-vivo efficacy of paromomycin in the *T. brucei brucei* STIB795 mouse model.

		Parasitemia positive			
Dose regimen per kg (i.p.)	Treatment period (days post infection)	Day 7	Day10	Day 14	Mean survival time (days)
Control^a^		4/4			6.5
4×100 mg	3–6	4/4			9.3
4×200 mg	3–6	3/4	4/4		13.5
10×100 mg	3–12	0/4	4/4		16.0
10×200 mg	3–12	0/4	0/4	4/4	22.0

a Untreated control used to determine the mean survival.

## Discussion

Protein synthesis is an established drug target in antibacterial chemotherapy and has been considered as target for antiprotozoal drugs [14]. Although the mechanisms of action of drugs targeting the ribosome have still to be studied in more detail, it has become clear that both cytosolic and organelle ribosomes represent potential drug targets [15], [16]. The plastidal ribosome is an established drug target in Apicomplexa [17], and serves as target for drugs such as clindamycin in the treatment of Toxoplasma [18] or plasmodia infections [19].

Among the various aminoglycosides paromomycin has been repeatedly found to be the most potent antiprotozoal compound. In early studies, paromomycin showed good antiamoebic and some antitrichomonal activity [20] whereas the related compound neomycin, which differs from paromomycin by a single substituent (amino instead of hydroxy group at position 6′; Figure S2), was poorly active against *Entamoeba histolytica* [21]. Likewise, paromomycin was shown to be more potent against amoebiasis than kanamycin, neomycin, and gentamicin, respectively, and in a study on *Giardia lamblia* paromomycin was the only 2-deoxystreptamine that showed activity [22]. Until recently, leishmaniasis was exclusively treated with sodium stibogluconate, pentamidine, or amphotericin B, but such treatments are expensive and potentially toxic. In contrast, paromomycin has been recently proposed as a well-tolerated and affordable treatment for visceral leishmaniasis (reviewed in [3]), which is still considered the second biggest parasitic killer after malaria.

Aminoglycoside antibiotics are cationic compounds with a 2-deoxystreptamine core that is glycosidically linked at position 4 to a glucopyranosyl (Fig. S2). Additional amino sugars are attached to either position 5 or 6 of the 2-deoxystreptamine moiety. Both the 4,5- and 4,6-disubstituted aminoglycosides target the ribosome by direct interaction with ribosomal RNA, affecting protein synthesis by inducing codon misreading and by inhibiting translocation of the tRNA-mRNA complex [23]–[25]. The binding site is defined by a number of nucleotides in the proximal stem of helix 44 in bacterial 16S rRNA (reviewed in [26]). Genetic and biochemical studies showed that the following bases are particularly relevant for aminoglycoside binding: A1408, C1409—G1491, and U1406—U1495 [12], [27]–[30].

We have previously emphasized the role of nucleotide 1408 in aminoglycoside susceptibility by studies of a cytosolic rRNA chimera of the trypanosome *Blastocrithidia*. This parasite carries a ribosomal decoding site that is identical to that of Leishmania and Trypanosoma with the exception of a 1408 adenosine instead of a guanosine [5]. In line with structural predictions, the cytosolic A-site of *Blastocrithidia* was found to be highly susceptible to all aminoglycosides tested. In contrast, the aminoglycoside target site in chimeric cytosolic ribosomes of *Leishmania* and *Trypanosoma* showed drug susceptibility merely to paromomycin but not to other aminoglycosides [5] (Fig. 2). These findings are in agreement with previous observations that *Leishmania* cultured in vitro is susceptible to paromomycin [31], [32]. Maarouf et al [8] have reported that paromomycin interferes with mitochondrial protein synthesis in *Leishmania*, whereas Horvath et al. [9] found no effect of paromomycin on mitochondrial protein synthesis. As a result, the precise role of mitochondria in trypanosome susceptibility to aminoglycosides has remained elusive.

The uracil-uracil opposition at position 1406—1495 is universally conserved across the three phylogenetic domains of life including their organelle ribosomes. The trypanosome mitochondrial ribosomes are exceptional in that they are characterized by a C—G base pair at 1406—1495. Any alteration of the U—U pair, particularly in U1406, results in decreased susceptibility to 4,6-substituted aminoglycosides such as gentamicin and tobramycin [26]. This is most likely due to a distortion of the amino sugar attached to position 6 of the aminocyclitol ring, thereby disrupting its hydrogen bonds to G1405. The conformation of aminoglycosides with an amino sugar attached to position 5 of the aminocyclitol ring is such that no contacts are made with G1405, and thus binding of 4,5-aminoglycsides is generally less affected by base alterations in 1406—1495. Hence, with regards to 4,6-substituted compounds, the 1406C—1495G pair confers high-level drug resistance (Fig. 2). It appears that the C–G pair alone renders the mitochondrial ribosome of trypanosomes resistant to a large number of aminoglycoside antibiotics, except the 4,5-disubstituted aminoglycosides paromomycin and neomycin.

Neomycin has significant activity against the trypanosome mitochondrial A-site in recombinant chimeric ribosomes. Among the aminoglycosides tested, which include paromomycin, gentamicin, and tobramycin, neomycin has the most potent antimitoribosomal activity (Fig. 2). However, in accordance with previous studies, we find that neomycin has no anti-trypanosomal activity in cell culture in-vitro (Table 1). Together with recent results demonstrating the essentiality of mitochondrial translation in the life cycle of *T. brucei*, our findings indicate that mitochondrial translation is not accessible to aminoglycosides. The organelles' resistance to neomycin action apparently is not due to an intrinsic resistance of the drug target structure, but most likely reflects limited mitochondrial permeability.

The observation that the cytosolic ribosomes of *Trypanosoma* are identical to that of *Leishmania* with respect to both rRNA sequence of the drug binding site and aminoglycoside susceptibility prompted us to test the efficacy of paromomycin against *Trypanosoma* in vitro and in vivo. Paromomycin was the most active antibiotic when compared to other aminoglycosides targeting protein synthesis. Intriguingly, in the cell culture model *T. brucei* showed significantly higher susceptibility to paromomycin than *Leishmania donovani*. In the *T. brucei* STIB795 mouse model, paromomycin treatment was able to suppress parasitemia and thus significantly increase the mean survival time of infected mice, suggesting that paromomycin inhibits the growth of *T. brucei*. However, paromomycin treatment did not eradicate the pathogen and relapse occurred in all tested animals after termination of treatment. Apparently, the anti-trypanosomal activity of paromomycin is sufficient to prevent *Trypanosoma* propagation and spread, but by itself is insufficient to cure the disease. Together, the findings reported here point to additional potential for antiprotozoal aminoglycosides beyond the currently established treatment options.

## Supporting Information

Figure S1 **Sequence alignments of** ***Leishmania*** **and** ***Trypanosoma*** **rRNA genes.** Nucleotides coding for the rRNA helices depicted in Figure 1 are highlighted in yellow. Nucleotides homologous to bacterial 16S rRNA residues 1408, 1492, and 1493 are colored in blue.
(PDF)

Figure S2 Chemical structures of the 2-deoxystreptamines used in this study.
(PDF)

Table S1 Plasmids used in this study.
(PDF)

Table S2 Strains used in this study.
(PDF)
