# Supplementary material for: Genetic Reconstruction of Protozoan rRNA Decoding Sites Provides a Rationale for Paromomycin Activity against Leishmania and Trypanosoma
Source: PLoS Negl Trop Dis. 2011 May 24;5(5):e1161. doi: 10.1371/journal.pntd.0001161 (PMC3101183; doi:10.1371/journal.pntd.0001161)
Supplement: Figure S1 — Sequence alignments of Leishmania and Trypanosoma rRNA genes. Nucleotides coding for the rRNA helices depicted in Figure 1 are highlighted in yellow. Nucleotides homologous to bacterial 16S rRNA residues 1408, 1492, and 1493 are colored in blue. (PDF) [file pntd.0001161.s001.pdf]

Figure S1. Sequence alignments of *Leishmania* and *Trypanosoma* rRNA genes. Nucleotides coding for the rRNA helices depicted in Figure 1 are highlighted in yellow. Nucleotides homologous to bacterial 16S rRNA residues 1408, 1492, and 1493 are colored in blue.

Leishmania 9S rRNA

```
L. donovani     526 AATAAATTTAGAAGGTATTGTTGCCCACCATTCTTTGTAATAAAGACAACGTGCAGTAATTAATGTATTTATAAAAATATATTTT 610
L. major        527 AATAAATTTAGAAGGTATTGTTGCCCACCATTCTTTGTAATAAAGACAACGTGCAGTAATTAATGTATTTATAAAAATATATTTT 611
L. amazonensis  526 AATAAATGTAGAAGGTATTGTTGCCCACCATTCTTTGTAATAAAGACAACGTGCAGTAATTAATATATTTATAAAAATATATTTT 610
L. tarentolae   527 AATAAATTTAGAAGGTATTGTTGCCCACCATTCTTTGTAATAAAGACAACGTGCAGTAATTAATATATTTATAAAAATATATTTT 611
```

Trypanosoma 9S rRNA

```
T. brucei  524 AAAATAAGAAATTAAAAAGGTATTGTTGCCCACCAATTTTTATAATAAAAATAACGTGCAGTAATTAATATATTTATAAAAATATATT 611
T. cruzi   520 AAAATAATAAATTCAAAAGGTATTGTTGCCCACCAATTTTTATAATAAAAATAACGTGCAGTAATCAATATATTTATAAAAATATATT 607
```

Leishmania and Trypanosoma 18S rRNA

```
L. donovani     2035 ACCGCCCGTCGTTGTTTCCGATGATGGTGCAATACAGGTGATCGGACAGGCG-GTGTTTTATCCGCCCGAAAGTTCACCGATATTTCTTCAATAGAGGAAGCAAAAGTCGTAACAA 2143
L. major        2035 ACCGCCCGTCGTTGTTTCCGATGATGGTGCAATACAGGTGATCGGACAGGCG-GTGTTTTATCCGCCCGAAAGTTCACCGATATTTCTTCAATAGAGGAAGCAAAAGTCGTAACAA 2143
L. mexicana     2036 ACCGCCCGTCGTTGTTTCCGATGATGGTGCAATACAGGTGATCGGACAGGCG-GTGTTTTATCCGCCCGAAAGTTCACCGATATTTCTTCAATAGAGGAAGCAAAAGTCGTAACAA 2144
L. amazonensis  2036 ACCGCCCGTCGTTGTTTCCGATGATGGTGCAATACAGGTGATCGGACAGGCG-GTGTTTTATCCGCCCGAAAGTTCACCGATATTTCTTCAATAGAGGAAGCAAAAGTCGTAACAA 2144
L. braziliensis 2036 ACCGCCCGTCGTTGTTTCCGATGATGGTGCAATACAGGTGATCGGACAGGCG-GTGTTTTATCCGCCCGAAAGTTCACCGATATTTCTTCAATAGAGGAAGCAAAAGTCGTAACAA 2144
L. tarentolae   2035 ACCGCCCGTCGTTGTTTCCGATGATGGTGCAATACAGGTGATCGGACAGGCG-GTGTTTTATCCGCCCGAAAGTTCACCGATATTTCTTCAATAGAGGAAGCAAAAGTCGTAACAA 2143
T. brucei       2070 ACCGCCCGTCGTTGTTTCCGATGATGGTGCAATACAGGTGATCGGACCGTCGCTCGTCTCGGGCGACCGAAAGTTCACCGATATTGCTTCAATAGAGGAAGCAAAAGTCGTAACAA 2189
T. cruzi        2099 ACCGCCCGTCGTTGTTTCCGATGATGGTGCAATACAGGTGATCGGACAGTCGAGTGCTTCACTTGACCGAAAGTTCACCGATATTTCTTCAATAGAGGAAGCAAAAGTCGTAACAA 2218
```
