# Supplementary figures and images for: Genetic Reconstruction of Protozoan rRNA Decoding Sites Provides a Rationale for Paromomycin Activity against Leishmania and Trypanosoma
Source: PLoS Negl Trop Dis. 2011 May 24;5(5):e1161. doi: 10.1371/journal.pntd.0001161 (PMC3101183; doi:10.1371/journal.pntd.0001161)

**Figure S2. Chemical structures of 2-deoxystreptamines used in this study.**

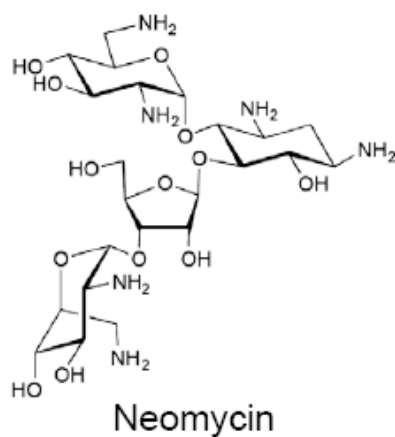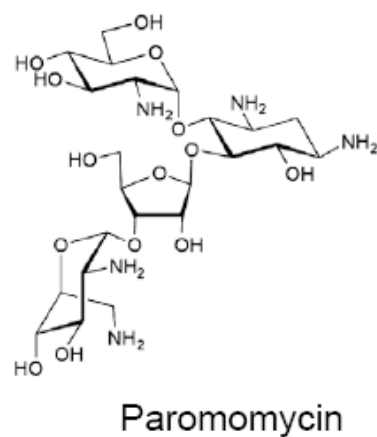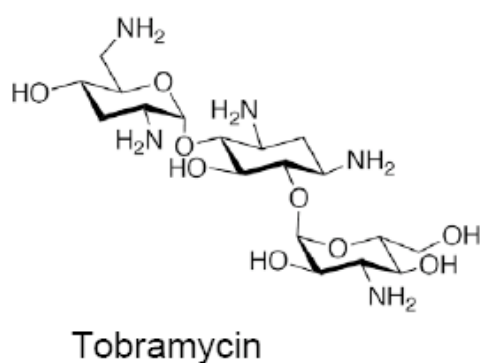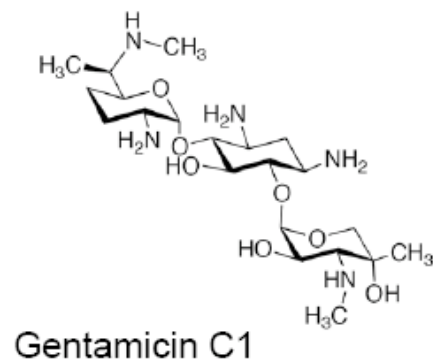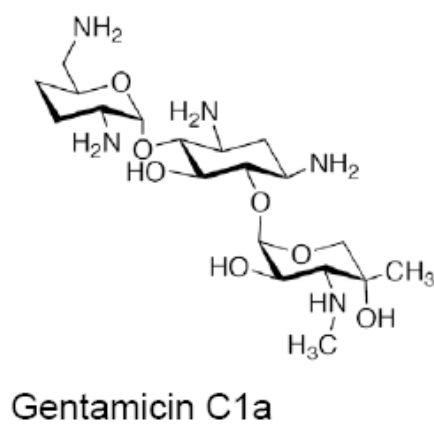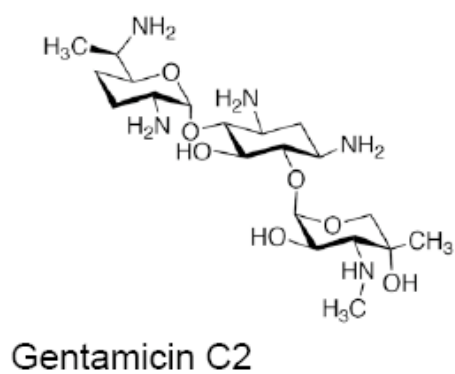

Supplement: Figure S2 — Chemical structures of the 2-deoxystreptamines used in this study. (PDF) [file pntd.0001161.s002.pdf]
