# Supplementary material for: Genetic Reconstruction of Protozoan rRNA Decoding Sites Provides a Rationale for Paromomycin Activity against Leishmania and Trypanosoma
Source: PLoS Negl Trop Dis. 2011 May 24;5(5):e1161. doi: 10.1371/journal.pntd.0001161 (PMC3101183; doi:10.1371/journal.pntd.0001161)
Supplement: Table S1 — Plasmids used in this study. (PDF) [file pntd.0001161.s003.pdf]

Table S1. Plasmids used in this study

Number	Plasmid	*marker*	A-site rRNA
pH144	pMIG-*rrnB*$^{+}$-*sacB*	Gm	Bacterial
pH150	pMIH-*rrnB*$^{+}$	Hyg	Bacterial
pH157	pMIH-*rrnB*(Leishm Cyt15)	Hyg	Leishmania cytosolic<br>Trypanosoma cytosolic
pH159	pMIH-*rrnB*(Leishm Mit13)	Hyg	Leishmania mitochondrial
pH160	pMIH-*rrnB*(Tryp Mit12)	Hyg	Trypanosoma mitochondrial
pZ154	pMIH-U1406C/U1495G	Hyg	Leishmania mitochondrial<br>Trypanosoma mitochondrial
