# Supplementary material for: Genetic Reconstruction of Protozoan rRNA Decoding Sites Provides a Rationale for Paromomycin Activity against Leishmania and Trypanosoma
Source: PLoS Negl Trop Dis. 2011 May 24;5(5):e1161. doi: 10.1371/journal.pntd.0001161 (PMC3101183; doi:10.1371/journal.pntd.0001161)
Supplement: Table S2 — Strains used in this study. (PDF) [file pntd.0001161.s004.pdf]

Table S2. Strains used in this study

M. smegmatis strain	Strain number	Parental strain	*attB*[a]	A-site rRNA
Δ*rrnB rrnA*$^+$	SZ004		wt	Bacterial
Δ*rrn* pMIG-*rrnB*$^+$-*sacB*	SZ558	SZ004	pH144	Bacterial
Δ*rrn rrnB*$^+$	SZ590	SZ558	pH150	Bacterial
Δ*rrn rrnB*(Leishm Cyt15)	SZ540<br>SZ542	SZ558	pH157	Leishmania cytosolic<br>Trypanosoma cytosolic
Δ*rrn rrnB*(Leishm Mit13)	SZ547<br>SZ550	SZ558	pH159	Leishmania mitochondrial
Δ*rrn rrnB*(Tryp Mit12)	SZ551<br>SZ554	SZ558	pH160	Trypanosoma mitochondrial
Δ*rrnB rrnA*(U1406C/U1495G)	SZ0337<br>SZ0338	SZ004	wt[b]	Leishmania mitochondrial<br>Trypanosoma mitochondrial

[a] rRNA hybrids were generated by replacing the wild-type rRNA genes in the *attB*-site of SZ558 with hybrid rRNA genes.
[b] The U1406C-U1495G mutant was generated by homologous recombination between the wild-type gene and introduced mutant sequence. Spontaneous plasmid curing after recombination restored the wild-type *attB* site.
